# MicroRNAs, Multiple Sclerosis, and Depression

**DOI:** 10.3390/ijms22157802

**Published:** 2021-07-21

**Authors:** Hsiuying Wang

**Affiliations:** Institute of Statistics, National Yang Ming Chiao Tung University, Hsinchu 30010, Taiwan; wang@stat.nctu.edu.tw

**Keywords:** biomarker, depression, microRNA, multiple sclerosis, treatment

## Abstract

Multiple sclerosis (MS) is a chronic disease of the central nervous system that affects the brain and spinal cord. There are several disease courses in MS including relapsing–remitting MS (RRMS), primary progressive MS (PPMS), and secondary progressive MS (SPMS). Up to 50% of MS patients experience depressive disorders. Major depression (MD) is a serious comorbidity of MS. Many dysfunctions including neuroinflammation, peripheral inflammation, gut dysbiosis, chronic oxidative and nitrosative stress, and neuroendocrine and mitochondrial abnormalities may contribute to the comorbidity between MS and MD. In addition to these actions, medical treatment and microRNA (miRNA) regulation may also be involved in the mechanisms of the comorbidity between MS and MD. In the study, I review many common miRNA biomarkers for both diseases. These common miRNA biomarkers may help further explore the association between MS and MD.

## 1. Introduction

Multiple sclerosis (MS) is a chronic and immune-mediated disease of the central nervous system. It is one of the most common causes of neurological dysfunction among young people worldwide. The incidence and prevalence of MS are increasing globally, even in areas of the world where the prevalence is traditionally low [1]. Comparative studies of different populations show that its prevalence and incidence rates vary with geographic location and ethnicity. The prevalence of MS ranges from 2 cases per 100,000 people in Japan to more than 100 cases per 100,000 in Northern Europe and North America [2]. It is a disabling disease that causes devastating economic and social impacts. The burden of MS is also affected by longevity and comorbidities.

There are different types of MS: relapsing–remitting MS (RRMS) and progressive MS, including primary (PPMS) or secondary (SPMS). Most MS patients are the RRMS type. They may have the first signs of the disease at an early age. After that, they relapse from time to time and then recover. Many patients with RRMS may move to the progressive phase. Because MS can affect any part of the central nervous system, its manifestations are usually diverse. Nevertheless, some key features may be clinically useful such as a variety of symptoms and signs that are involved in motor, sensory, visual, and autonomous systems [3]. The most common manifestations are optic neuritis, and brainstem and spinal cord syndrome. In addition, there are many other less common manifestations, including cortical manifestations. The recurrence of MS may last for several weeks to reach a plateau, and then patients gradually recover. Most relapses can cause damage [4]. 

The risk of MS is significantly different between different races. Several studies have shown that the risk factors of MS include genetic and environmental factors.

The environmental risk factors of MS including latitude of habitation, vitamin D, lifestyle, viruses, and other infectious agents [5]. It was demonstrated that the serum levels of vitamin D were reduced in MS patients, and the role of vitamin D in the pathogenesis of MS has been fully confirmed [6,7]. The fact that lower vitamin D contributes to the prevalence of MS may explain the connection between the latitude of habitation and the risk of developing MS. It was studied that the majority of MS cases lived in regions where sunlight is less intense [8]. In addition, smoking, obesity, diet, changes in the gut microbiome, and exposure to industrial chemicals might increase the risk of developing MS [5]. Endogenous retrovirus reactivation and Epstein Barr virus (EBV) infection were associated with MS [5]. So far, there is no effective treatment for MS. Several approved therapies including glatiramer acetate, interferon-β (IFN-β), and mitoxantrone mainly target the immunological aspects of MS [9].

Major depression (MD) is a serious mood disorder and comorbidity of many diseases, including MS. The symptoms of MD may include the feeling of hopelessness, fatigue, loss of interest in normal activities, impaired concentration, sleep disturbances, recurring thoughts of death or suicide, and anxiety. The number of people suffering from MD continues to increase over time. MD can be diagnosed with psychological tests that can measure the severity of MD by asking participants personal questions. In addition to MS, MD was shown to be associated with several other diseases such as obesity, diabetes, cancer, stroke, and acute coronary syndrome [10,11,12,13]. MD was also associated with gastroesophageal reflux disease [14]. The gut microbiome in the gastrointestinal tract was a risk factor for the development of MD and the persistence of depressive symptoms [15].

About half of patients with MS experience depressive disorders [16]. Patients with depressive symptoms have a worse prognosis than those without. MD also is an important factor causing higher suicide rates of MS patients [17]. Some immunomodulatory drugs for MS such as IFN-β have been shown to link to MD [18]. IFN-β was shown to reduce the recurrence rate and delay physical disability in RRMS and SPMS [19]. MS patients often experience typical depression symptoms such as pain, fatigue, and cognitive impairment. So far, the genetic pathogenesis of the relationship between MS and MD is still unclear [20]. In this study, I explore the common microRNA (miRNA) biomarkers of MS and MD. Because MS and MD share many common miRNA biomarkers, the relationship between MD and MS will be discussed from the aspect of miRNA biomarkers. 

## 2. MicroRNA

miRNA is a small non-coding RNA with a length of about 21–24 nucleotides. It has important functions in cell differentiation, development, cell cycle regulation, and apoptosis. miRNAs can regulate up to 30% of the protein-coding genes in the human genome [21], and it is well known that they are involved in the development of many diseases. Strong evidence revealed that in cancer cells, miRNAs were dysregulated due to various mechanisms including abnormal transcriptional controls of miRNAs, dysregulated epigenetic changes, and defects in the miRNA biogenesis pathway [22]. As a result, miRNAs are good biological biomarkers of various cancers, and many bioinformatics tools have been developed to predict miRNA biomarkers of cancers [23,24,25,26,27,28]. 

One of the main barriers to cancer chemotherapy is the drug resistance problem. miRNAs were also shown to contribute to the development of resistance against chemotherapy [29]. In addition to cancers, abnormal miRNA expression also contributes to neurological and psychiatric diseases such as frontotemporal dementia, Alzheimer’s disease, Parkinson’s disease, spinal muscular atrophy, amyotrophic lateral sclerosis, and anti-NMDA receptor encephalitis [30,31,32,33,34,35]. miRNA biomarkers have been used to explore the association between different diseases and the association between vaccination and diseases [35,36,37]. 

In the therapeutic aspect, the development of small RNA drugs has significantly progressed. The first small-interfering RNA (siRNA) drug, patisiran, was approved by the Food and Drug Administration (FDA) in 2018 [38]. Another siRNA-based drug, givosiran, was also approved by the FDA in 2019 [39]. So far, compared with siRNA-based drugs, there has been less advance of miRNA-based drugs. The slow development of miRNA therapeutics might be due to too many targets for the miRNA effects, which might cause miRNA-based drugs to trigger a series of unpreventable consequences [39]. Nevertheless, there is still potential to develop miRNA-based drugs, and there have been 10 miRNA-based drugs in clinical trials [39]. Exosomes are nano-sized bio-vesicles released from the endocytic compartment of cells into surrounding body fluids, such as serum. Bovine milk-derived exosomes were shown to be a promising source as a nanocarrier of miRNAs for RNA-based therapy [40]. 

## 3. MicroRNA Biomarkers

In this study, I discuss the common miRNA biomarkers of MS and MD. Many common miRNA biomarkers of both diseases are presented in Table 1. Nevertheless, there may be more common miRNA biomarkers of MS and MD than those listed in Table 1.

Most of the miRNAs in Table 1 are reviewed as follows. The miRNA expression was obtained from the peripheral blood mononuclear leukocytes from 10 Chinese MS patients and 10 healthy controls [41]. Then the study was validated independently using real-time polymerase chain reaction (PCR) in the second cohort of 40 MS patients and 40 controls. The levels of miR-125a, miR-146b, and miR-200c were elevated in these MS patients, whereas miR-328, miR-199a, and miR-152 were decreased. The active lesions in the brains of the early stages of MS patients contain many inflammatory cells and macrophages. Moreover, the cerebrospinal fluid (CSF) of MS patients bearing active demyelinating lesions had abnormally high miR-125a-3p levels [42]. Interleukin 17 (IL-17)-producing T helper cells (TH-17 cells) were shown to be implicated with MS [122]. miR-326 was associated with the pathogenesis of MS by regulating TH-17 differentiation [62]. The protein urocortin 1 (Ucn1) is most abundantly expressed in the midbrain, and depressed suicide completers have upregulated midbrain Ucn1 expression levels compared with control individuals [123]. miR-326 acted as a molecular switch in the regulation of midbrain Ucn1 expression [65]. A DNA methylation analysis was performed in CD4+ T cells from RRMS, SPMS, and healthy individuals [69]. RRMS patients had lower levels of miR-21 compared to SPMS patients and healthy individuals. Ahmadian-Elmi et al. compared 40 RRMS patients including 20 samples in relapsing and 20 samples in remitting phases with the control group [71]. miR-27a was upregulated in the relapsing phase compared to the remitting phase and healthy individuals, while miR-214 was downregulated in the relapsing phase compared to remitting phase and healthy individuals. miR-15a was downregulated in CD4+ T cells from RRMS patients [75]. miR-320a and miR-125a-5p were significantly upregulated in pediatric MS and adult-onset MS patients [43]. miR-184 could promote oligodendrocyte differentiation that was involved in developing a cell-based therapy for MS [81]. miR-23b, which could halt the progression of experimental autoimmune encephalomyelitis (EAE), was a potential therapeutic target in the amelioration of MS [87]. 

Elevated levels of miR-181c were observed in the CSF of MS patients [88]. miR-340 expression in memory CD4+ T-cells increased in MS patients [90,91]. miR-629 was upregulated, but miR-30a-3p and miR-497 were downregulated in CD8+ T cells from peripheral blood samples of RRMS patients [93]. Some differentially expressed miRNAs obtained from several studies were validated in 86 MS patients and 55 controls [55]. miR-328 and miR-30a were upregulated in MS patients, and miR-21, miR-199a, miR-365, and miR-146a were downregulated in MS patients compared with controls. Bioinformatics analysis revealed that miR-532-5p was differentially expressed in RRMS patients, and a digital quantitative PCR method confirmed the downregulation of exosomal miR-532-5p in RRMS relapse patients [98]. Compared with 20 healthy controls, miR-126-5p was upregulated in 17 RRMS patients [100]. Longitudinal analysis revealed that miR-18a and miR-20b were upregulated and predominantly expressed in CD4+ T cells from RRMS patients, whereas miR-326 was downregulated upon natalizumab treatment. An upregulation of miR-9-5p in the relapsing phase of 40 MS patients was observed compared with 11 healthy controls that suggested a possible inducing role of miR-9-5p in the pathway of Th17 cells during MS pathogenesis [104]. MS was characterized by the demyelination of central nervous system neurons. miRNAs play a role in remyelination, contributing to MS, including miR-219, miR-338, miR-125a, miR-27a, miR-146a, miR-9, miR-23, miR-184, miR-124, miR-223, miR-155, miR-30a, miR-34a, miR-326, and miR-27. Compared with the baseline, let-7c and miR-125a-5p were decreased, while miR-642 was increased after 6 and 12 months of the natalizumab therapy [46]. miR-142-5p, miR-199a-5p, and miR-330-3p showed a significant difference between MS patients and controls in terms of the expanded disability status scale score [58]. TaqMan array analysis showed that miR-126, miR-193a, and miR-486 were significantly increased, whereas miR-34a was decreased in CD4+ T cells from peripheral blood mononuclear cells of RRMS patients [73]. miR-124, miR-486a, and miR-532 were significantly decreased in the nucleus accumbens of chronic unpredictable mild stress-induced mice with depression-like behaviors, while miR-388 was significantly upregulated [99]. Exosomal miR-15b-5p and miR-451a were differentially expressed in 25 RRMS patients compared with 11 controls [110]. let-7b-5p is negatively associated with inflammation and disease severity in MS [118]. Upregulation of both miR-326 and miR-26a in the relapsing phase of MS patients compared with remitting phase and healthy controls was observed [64]. Let-7b, miR-20b, miR-30a, miR-125a, miR-146a, miR-221, and miR-328 were differently expressed in MS patients [47].

The miRNA profiles from active and inactive MS lesions were established by PCR in 16 active and 5 inactive white matter MS brain lesions and 9 control white matter specimens [52]. miRNAs were found at least twice more abundant or less abundant than in normal white matter in inactive lesions (see miRNAs of reference [52] in Table 1). Monocytes–macrophages were shown to influence the inflammatory activity and demyelination in MS. The expression of miRNAs impacting monocyte–macrophage immune function and their communication with brain cells in MS patients was investigated [44]. The levels of miR-146a, miR-223, miR-125a, and miR-30c were increased in both RRMS and PPMS patients compared with controls, and the level of miR-155 was decreased in both PPMS and RRMS patients compared with controls. In addition, reduced levels of miR-124 were observed in PPMS patients compared with controls and RRMS patients. 

Blood samples from 40 RRMS patients and 20 healthy volunteers were investigated [57]. The expression levels of miR-34a, miR-199a, miR-30c, and miR-19a, and the percentage of Th17 and Treg cells were measured. An increased expression of miR-34a, miR-30c, and miR-19a in the relapsing phase and a decreased expression of miR-199a in the remitting phase were observed. A correlation was shown between the four miRNAs, miR-34a, miR-199a, miR-30c, and miR-19a, with different phases of MS, and these miRNAs were involved in differentiation pathways of Th17 cells. The association between the miRNA expression level in CSF and gadolinium-enhancing (Gd+) lesions was investigated to identify the miRNA biomarkers of MS [109]. A total of 28 miRNA candidates in CSF collected from 46 patients with MS (26 Gd+ and 20 Gd− patients) were performed by TaqMan assays and PCR. Increasing levels of miR-21 and miR-146a/b were observed in Gd+ MS patients. miRNAs related to treated MS patients were studied [66]. Eleven RRMS patients were classified into two groups: four untreated patients who had received no treatment with any disease-modifying therapy, and seven treated patients with the immunomodulatory drug IFN-β with at least a 2-year follow-up. PCR analysis showed that 16 miRNAs were differentially expressed in the two groups (see miRNAs of reference [66] in Table 1). 

Lithium (Li) is commonly used in the treatment of bipolar disorder known as manic depression. miR-34a, miR-152, miR-155, and miR-221 were consistently upregulated in 20 lymphoblastoid cell lines with Li treatment at treatment time points day 4 and day 16 [60]. The glutamate receptor, metabotropic 4 (GRM4), that can regulate MD was an attractive target for drug discovery [54]. To investigate GRM4 regulation, an analysis showed that miR-650 and miR-328 were downregulated and upregulated in the blood samples of 18 MD patients compared with healthy controls, respectively [54]. miR-21 was reduced in the white matter of mice with MD [70]. miRNA-15a was increased in the amygdala–Ago2 complex in the mice exposing to chronic stress [76]. β-catenin has been implicated in MD. miR-214-3p was significantly upregulated in the medial prefrontal cortex of chronic social defeat stress mice by targeting β-catenin transcripts [80]. Brain-enriched miRNA-184 is downregulated in older patients with MD [82]. MD patients had significantly increased exosomal miR-139-5p levels when compared with controls [85]. Serum expression levels of miR-23b-3p and miR-142-3p significantly increased in 102 bipolar II disorder patients compared with 118 controls [68]. Acute stress could induce abnormal expressions of miR-9, miR-30c, miR-125a, miR-126, and miR-487b in mice [49]. In a rat model study, rats were exposed to repeated inescapable shocks and tested for learned helplessness [83]. miR-184, miR-125a, miR-181c, and miR-18a were implicated in stress and depression. A machine learning analysis using 33 miRNAs to distinguish MD cases and controls could attain high accuracy [72]. These miRNAs that could distinguish MD cases and controls included miR-27a, miR-22, miR-221, miR-126, miR-148a, miR-155, miR-140, miR-629, and miR-28. miR-139-5p and miR-195 were significantly upregulated in the prefrontal cortex of suicides with a history of MD compared with healthy controls [86]. Androgen receptor (AR) and stress might influence the development of MD. Decreased AR might accelerate the stress-induced MD by changing miR-204-5p/BDNF/AKT/MAPK signaling [96]. 

miR-30a levels were significantly upregulated in MD patients exposed to childhood trauma [97]. The role of miRNAs was examined in chronic corticosterone-mediated depression rats [54]. Chronic corticosterone administration to rats caused altered expression of miRNAs in the prefrontal cortex including upregulated miRNAs, miR-124, miR-181c, and miR-365, and downregulated miRNAs, miR-146a, miR-200c, miR-155, miR-135a, and miR-487b. Novel miR-213 was upregulated in depression-like mice [108]. Peripheral blood miRNAs altered in MD people compared to controls, including upregulated miRNAs, miR-451, miR-328, miR-199a, miR-330, miR-125a, miR-30a, miR-34a, and miR-155 and downregulated miRNAs, miR-320a, miR-532, miR-650, and miR-15b [51]. The expression of miR-19a-3p might be influenced by strong suicidal thoughts in MD patients, and it was upregulated in individuals who had committed suicide [116]. miR-122 was observed to be a promising miRNA candidate for a diagnostic biomarker of MD, with a significant decrease in MD cases [74]. miR-215-5p showed significant upregulation in 15 MD subjects compared with 15 controls [120]. The expression levels of miR-221 in the CSF and serum of MD patients was increased as well as in the hippocampus of chronic unpredictable mild stress mice [124].

## 4. Other Mechanisms Connecting MS and MD

In addition to common miRNA biomarkers that may contribute to the comorbidity between MS and MD, the connection between the two diseases can be discussed from other aspects. The immunomodulatory drug IFN-β for MS was reported to increase depressive symptoms in patients. Interferons (IFNs) are a group of signaling proteins known as cytokines. IFNs are released by host cells to defend the presence of viruses and can be used to compete against infections and immune responses such as in the treatment of autoimmune, viral, and malignant disorders. There are several different types of IFNs according to their receptors. IFN-α was the first cytokine to be approved to treat hairy cell leukemia in 1986 [125]. Receptors for IFN-β are present on all cells in the human body. IFN-β has several functions that can treat MS such as inhibiting proinflammatory cytokines IL-17, increasing anti-inflammatory agents IL-10 and attenuating leukocyte migration across the blood-brain barrier [126]. However, IFN therapy has troublesome adverse effects [127]. The incidence rate of the depression adverse effect of IFN-β in MS patients is greater than 0.1 [128]. A higher dose and longer duration of IFN treatment may increase the risk of IFN-related depression. 

INF-related depression may be caused by the interaction between immune, endocrine, and neuronal pathways. Pinto and Andrade explained this mechanism as “IFN therapy (α, β) induces hypothalamo–pituitary–adrenal axis hyperactivity to release corticotropin releasing hormone (CRH) from the median eminence of the pituitary gland. CRH increases adrenocorticotropic hormone and hence adrenal corticosterone release. CRH also decreases serotonin and noradrenaline in the paraventricular nucleus, prefrontal cortex, hippocampus, and central amygdala. These neuroendocrine and neurotransmitter changes are conventionally associated with risk of depression” [127].

In addition to the drug factor for MS that might induce MD, MS and MD patients share many other abnormalities including neuroinflammation, peripheral inflammation, gut dysbiosis, chronic oxidative and nitrosative stress, neuroendocrine abnormalities, and mitochondrial dysfunction [129]. Microglia account for approximately 10–15% of all cells in the brain. Activated microglia that might be caused by activated encephalitogenic Th17 T cells is a core of MS pathology. Activated microglia also contributed to MD [130]. Gut microbes were shown to connect many brain disorders [131,132]. Specific microbes could trigger the differential Th17 T-effector phenotype in mice studies [133]. Gut microbiota and dietary interventions have been suggested as promising treatments for MS [134]. In addition, MD patients have demonstrated gut microbiome dysbiosis [135]. As a result, gut microbiota modulation is a prospective intervention for the management of MS and MD. 

RRMS patients have significant peripheral levels of oxidative stress, and the oxidative and nitrosative stress might be a major factor driving the pathophysiology of MD [129]. Mitochondrial respiratory chain deficiency and abnormalities in mitochondrial transport were demonstrated in MS that might contribute to progressive neurodegeneration and irreversible disability of MS [136]. MD pathophysiology might be related to the impairment of neuroplasticity. Mitochondria play a key role in calcium homeostasis that is involved in the regulation of neurotransmission and neuroplasticity in the brain [137]. As a result, mitochondrial dysfunction also plays an important role in MD. 

## 5. Discussion

**Figure 1:**
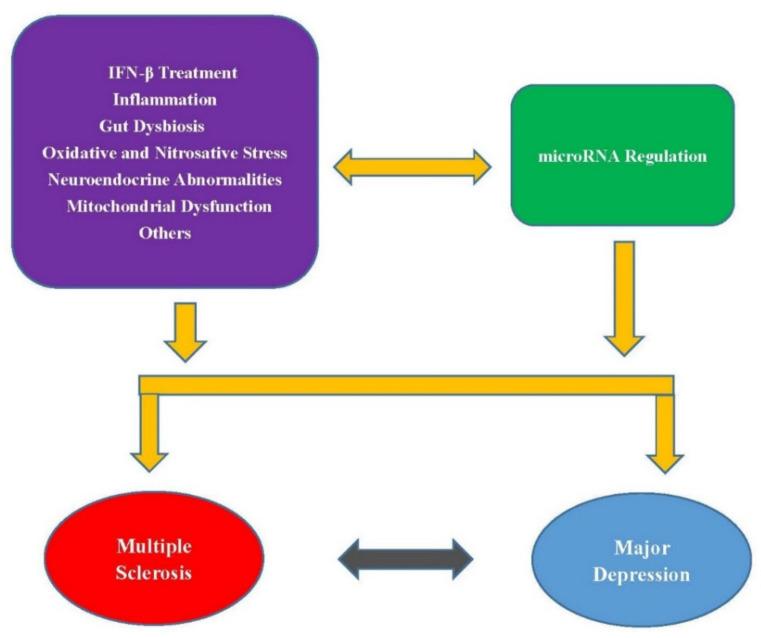
Possible abnormalities and treatment that directly link MS and MD or regulate common miRNA biomarkers of both diseases.

As mentioned above, there are many common biological actions as well as medical treatments involved in the comorbidity between MS and MD. These mechanisms may also be involved in miRNA regulation (Figure 1). Therefore, exploring common miRNA biomarkers of both diseases may help understand their connection pathology and develop treatments to reduce the depression symptoms in MS patients. Moreover, many miRNAs have been discussed to be used as circulating biomarkers for MS or MD. In addition to the miRNAs listed in Table 1, Martinez and Peplow reviewed many miRNAs in peripheral blood, serum, exosomes isolated from serum, and CSF that had altered expression levels in MS compared to controls [138]. Gheysarzadeh et al. identified the serum-based miRNAs, miR-16, miR-135a, and miR-1202, as miRNA biomarkers for MD [139]. However, further studies are needed to validate whether these miRNAs in serum or CSF can be used as potential diagnostic markers for MS or MD and for monitoring disease progression and response to therapy.

In addition, exosomal miRNAs were identified to be useful biomarkers of MS and MD. miRNAs can be found in peripheral blood, serum, exosomes, CSF, and tissues.

Some of the miRNAs discussed in Table 1 are exosomal miRNAs. Moreover, Mycko and Baranzini reviewed recent advances in miRNA and exosome profiling in MS [140]. Circulating exosomes were shown to be promising candidate biomarkers for MS. Exosome-associated miRNAs in serum samples from MS patients and healthy controls were profiled using small RNA next-generation sequencing [110]. Nine miRNAs, namely miR-15b-5p, miR-23a-3p, miR-223-3p, miR-374a-5p, miR-30b-5p, miR-433-3p, miR-485-3p, miR-342-3p, and miR-432-5p, were identified to distinguish RRMS from progressive MS. Liang et al. recruited 30 MD patients and 30 healthy control individuals to detect serum exosomal miR-139-5p levels [85]. MD patients had significantly increased exosomal miR-139-5p levels when compared with controls. In this study, I explored the common miRNA biomarkers for MS and MD, but not limited to exosomal miRNAs. Compared with other studies that only focus on miRNA biomarkers for MS or MD, this study can provide more useful knowledge in connecting MS and MD from the miRNA aspect. 

## 6. Conclusions

miRNAs are involved in the pathogenic mechanisms of many disorders. In this study, I review many common miRNA biomarkers of MS and MD. These two disorders share more common miRNA biomarkers than those discussed in this paper. It is known that psychiatric comorbidity is common in MS patients, and patients experiencing depression symptoms have a worse prognosis compared with those without depression symptoms. Because a large proportion of MS patients also have depression symptoms, understanding the pathologic linking of both diseases can help develop medicines to treat both diseases. 

Therefore, investigating the common miRNA biomarkers of MS and MD may be a useful direction to develop joint drugs to treat both diseases. The development of small RNA drugs such as siRNA and miRNA has progressed. There is potential to develop joint miRNA-based drugs for treating comorbidity diseases such as MS and MD. In addition to related miRNA mechanisms, neuroinflammation, peripheral inflammation, gut dysbiosis, chronic oxidative and nitrosative stress, neuroendocrine abnormalities, and mitochondrial dysfunction may contribute to the comorbidity of both diseases. These mechanisms may also contribute to MS and MD by regulating common miRNA biomarkers of both diseases. Therefore, the common miRNA biomarkers discussed in this paper may help further investigate the pathologic connection between MS and MD. 

## Figures and Tables

**Table 1:** The common miRNA biomarkers of MS and MD are provided. The expression information with parentheses means the expression information after the treatment of the drug.

miRNA	MS miRNA Expression	MS References	MD miRNA Expression	MD References
miR-125a	↑, ↓(Natalizumab)	[41,42,43,44,45,46,47]	↓,↑, ↓(Escitalopram)	[48,49,50,51]
miR-146b	↑	[41,52]	↓(Escitalopram), ↑(Duloxetine)	[48] [53]
miR-200c	↑	[41,52]	↓	[54]
miR-328	↓,↑	[41,47,52,55]	↑	[51,56]
miR-199a	↑,↓	[41,52,55,57,58]	↑	[51,59]
miR-152	↓,↑	[41]	↑(Lithium)	[60]
miR-650	↑	[52,61]	↓	[51,54]
miR-326	↑, ↓(Natalizumab)	[42,52,62,63,64]	↓	[65]
miR-142	↑,↓	[52,58,66]	↓,↑	[67,68]
miR-21	↑,↓	[52,69]	↓	[70]
miR-27a	↑	[45,52,71]	↓	[48,67,72]
miR-193a	↑	[52,73]	↓	[74]
miR-15a	↑	[52,75]	↑	[76]
miR-130a	↑	[52]	↓	[67]
miR-22	↑	[52,66]	↓,↑(Escitalopram)	[72,74,77]
miR-320	↑	[43,52,78]	↓	[51,79]
miR-214	↑,↓	[52,71]	↑	[80]
miR-184	↓	[45,52,81]	↓,↑	[50,82,83]
miR-139	↓	[52,61]	↑	[51,84,85,86]
miR-23b	↓	[52,87]	↑	[68]
miR-487b	↓	[52,61]	↑	[49,54]
miR-181c	↓	[52,88,89]	↓,↑	[50,54,83]
miR-340	↓,↑	[52,90,91]	↓	[92]
miR-629	↑	[52,93]	↑,↑(Escitalopram)	[72,74,77]
miR-148a	↑	[52,90]	↑	[72,94]
miR-28	↑	[52]		[72]
miR-195	↑	[52]	↑	[86]
miR-497	↑,↓	[52,73,93]	↓	[67]
miR-135a	↑	[52,61,95]	↓	[54,92]
miR-204	↑	[52,61,95]	↑	[96]
miR-660	↑,↓	[52,61,66,95]	↓	[67]
miR-30a	↑,↓	[45,47,52,55,61,93,95]	↑	[51,97]
miR-365	↑,↓	[52,55]	↑	[54]
miR-532	↑,↓	[52,98]	↓,↑(Escitalopram)	[48,51,99]
miR-126	↑	[52,73,100]	↓,↓(Escitalopram)	[48,49,72]
Let-7c	↑, ↓(natalizumab)	[46,52,61,95]	↓	[101]
miR-20b	↑,↓	[47,52,63]	↓	[67]
miR-30d	↑	[52,102]	↑,↑(Escitalopram)	[77,103]
miR-9	↑	[45,52,104]	↓	[49]
miR-219	↓	[45,52]	↓	[105]
miR-338	↓	[45,52]	↑	[99]
miR-642	↓, ↑(natalizumab)	[46,52]	↑	[67]
miR-181b	↓	[52,106]	↓(Escitalopram)	[48]
miR-18a	↓	[52,63]	↑,↑(Duloxetine)	[50,83,107]
miR-190	↓	[52,61]	↓	[67]
miR-213	↓	[52,61]	↑	[108]
miR-330	↓	[52,58]	↑	[51,59]
miR-151	↓	[52,61]	↓(Escitalopram)	[48]
miR-140	↓	[52,61]	↑(Escitalopram)	[77,83]
miR-146a	↑,↓	[44,45,47,52,55,66,109]	↓, ↑(Duloxetine), ↓(Escitalopram)	[48,53,54,67]
miR-223	↑,↓	[44,45,52,110]	↑,↓(Escitalopram)	[48,51]
miR-30c	↑	[44,57]	↓	[49,111]
miR-155	↑,↓	[44,45,52]	↑,↓,↑(Lithium)	[51,54,60,67,72]
miR-124	↓	[44,45]	↑,↓,↓(Duloxetine)	[54,99,107,112,113,114]
miR-34a	↑,↓	[45,52,57,73]	↑,↑(Lithium)	[51,60,115]
miR-19a	↑,↓	[57,66]	↑	[116]
miR-21	↑,↓	[55,109]	↑,↓	[70,74]
miR-22	↑	[66,117]	↓,↑(Escitalopram)	[74,77]
miR-486	↓	[66,73]	↓	[99]
miR-451a	↓,↑	[66,110]	↑	[51,79]
let-7b	↓,↑	[47,66,118]	↓	[101]
miR-320b	↓	[66,119]	↓	[74]
miR-122	↓,↑	[61,66,95]	↓	[74]
miR-215	↓	[66,106]	↑	[120]
miR-26a	↓,↑	[64,66]	↑(Escitalopram), ↓(Escitalopram)	[48,77]
miR-15b	↓	[66,110]	↑,↓	[51,121]
miR-221	↑	[47,106]	↑	[72]
